# Effect of bioactive compounds released from Brassicaceae defatted seed meals on bacterial load in pig manure

**DOI:** 10.1007/s11356-021-14321-7

**Published:** 2021-06-30

**Authors:** Luisa Ugolini, Donatella Scarafile, Roberto Matteo, Eleonora Pagnotta, Lorena Malaguti, Luca Lazzeri, Monica Modesto, Alice Checcucci, Paola Mattarelli, Ilaria Braschi

**Affiliations:** 1 CREA Council for Agricultural Research and Economics - Research Centre for Cereal and Industrial Crops, Via di Corticella 133, 40128 Bologna, Italy; 2 Department of Agricultural and Food Sciences, Alma Mater Studiorum Università di Bologna, Viale G. Fanin 44, 40127 Bologna, Italy

**Keywords:** Animal waste, Brassicas, Myrosinase, Glucosinolates, Isothiocyanates, *Escherichia coli*, *Staphylococcus aureus*, *Enterococcus faecalis*

## Abstract

Animal manure application to soils is considered to be one of the main cause of antibiotic and bacterial pathogen spread in the environment. Pig livestock, which is the source of one of the most used fertilizer for cultivated land, is also a hotspot for antibiotics and antibiotic-resistant bacteria. Besides harsh chemical and physical sanitization treatments for the abatement of antibiotics and bacterial load in livestock waste, more sustainable and environmentally friendly strategies need to be considered. In this context, the use of natural substances which are proved useful for pest and disease control is currently under exploration for their role in the reduction of bacterial pathogen population. Among these, plants and derived products from the Brassicaceae family, characterized by the presence of a defensive glucosinolate-myrosinase enzymatic system, have been successfully exploited for years in agriculture using the so-called biofumigation technique against crop diseases. Although the application of biofumigation to suppress a range of soil borne pests has been well documented, no studies have been examined to reduce bacterial population in animal waste. In the present study, the release and the antibacterial activity of bioactive compounds deriving from different Brassicaceae defatted seed meals against pathogens and bacterial population in pig manure is addressed. *Rapistrum rugosum* and *Brassica nigra* defatted seed meals were found to be the most active products against tested pathogens and able to significantly reduce the bacterial load in the manure.

**Supplementary Information:**

The online version contains supplementary material available at 10.1007/s11356-021-14321-7.

## Introduction

Agricultural application of animal manure as a soil amendment and crop fertilizer is a common practice that has been used since ever. Currently, the increasing livestock manure production due to the diffusion of intensive animal farming has to be carefully considered in term of possible negative effects on environmental ecosystems as well as animal and human health. Improper manure storage and management need to be considered in terms of soil organic matter and nutrient balance but also of pathogen load. Indeed, livestock manure is considered one of the main cause of bacterial pathogen and antibiotic spread in the environment, posing a risk for food and water contaminations. This is because of huge application of antibiotics for therapeutic or prophylactic use in such breeding farm. The role of manure in bacterial pathogen and antibiotic resistance bacteria dissemination (Checcucci et al. 2020) prompted further studies on possible treatments able to decrease bacterial load prior to land application. Several physical, chemical, and biological treatments may be employed to reduce pathogen population in livestock manure management (Bilotta and Kunz 2013). Meanwhile, the identification and development of techniques that are sustainable and economically feasible are required as the use of natural compounds with biological activities. In this context, the use of Brassicaceae biomasses as defatted seed meals (DSMs), by-product of seed oil extraction, could be a promising alternative to control pathogen spread.

Brassicaceae (order Brassicales) are a family of about 350 plant genera and 3700 species, including some edible and industrial oilseed crops of great economic importance (Blažević et al. 2020). Plants in the Brassicaceae family are characterized by the presence of a defensive endogenous system, the glucosinolate-myrosinase system (GSL-MYR), particularly concentrated in the seed but also active at the root system (McCully et al. 2008) and at the rhizosphere (Braschi et al. 2011; Braschi et al. 2008). GSLs, the main secondary metabolites found in Brassicaceae, are β-thioglucoside *N*-hydroxysulfates, which contain a sulfur-linked β-d-glucopyranose moiety and a variable hydrophobic aglycone side chain, derived from an α-amino acid precursor. More than 130 GSLs have been fully or partially characterized and are classified as aliphatic, thiofunctionalized, aromatic, or indolic GSL, according to the aglycone side chain structure (Agerbirk and Olsen 2012). GSLs co-occur in plant with the endogenous enzyme myrosinase, MYR (thioglucoside glucohydrolase, EC 3.2.1.147), but they are separately stored in GSL-containing S-cells and in the myrosin cells respectively (Andréasson et al. 2001). However, upon abiotic or biotic stress and consequent tissue disruption, GSLs and MYR come into contact, triggering, in presence of water, GSL hydrolysis. Different products could be formed in function of GSL chemical structure, reaction conditions (pH in particular), or the presence of specifier proteins (Hanschen et al. 2014). Isothiocyanates (ITCs) are generally the main reaction products formed at neutral pH. ITCs are bioactive compounds largely known for their broad-spectrum biological activity against nematodes, soil-borne fungi, and insects or weeds (Curto et al. 2016; Lazzeri et al. 2013; Matteo et al. 2018; Matthiessen and Kirkegaard 2006). The ITC release from brassica plants and derived products has been successfully exploited for years in agriculture using the so-called biofumigation technique for pest and disease control, as an alternative strategy to the use of synthetic pesticides (Müller et al. 2018; Ntalli and Caboni 2017; Ugolini et al. 2019). Furthermore, ITCs are known also for their healthy effect in human as they showed anticancer, anti-inflammatory, antioxidant but also antibacterial and antiviral activities, in in vitro and in vivo studies (Borges et al. 2014; Frankel et al. 2016; Mithen and Ho 2018; Quirante-Moya et al. 2020; Sikorska-Zimny and Beneduce 2020). Although the biocidal activity of ITCs against plant pathogens and pests is well documented, their activity against human/animal bacteria has received minor attention. Some studies showed the in vitro ITC effect against important human pathogens, including bacteria with resistant phenotypes, even if their mechanism of action at molecular level has not being completely clarified yet (Romeo et al. 2018). The high electrophilic ITC group could react with amine, thiol, or hydroxyl groups; thus, it may influence activities and functions of several molecular targets in bacterial cells (Dufour et al. 2015; Zhang 2012).The ITC antibacterial activity seems to have a multi-targeted mechanism of action in different bacterial strains, affecting several metabolic pathways, probably involving membrane damage, inhibition of enzyme activities, such as those of cellular respiration, induction of heat-shock and oxidative stress responses, and free amino acids depletion (Dufour et al. 2015; Nowicki et al. 2019; Romeo et al. 2018). Aromatic ITCs often showed higher activity than aliphatic ones. Based on results obtained by in vitro test with different ITCs, some authors have postulated that phenyl rings could be a predisposing factor for antibacterial efficacy (Dias et al. 2012; Kim and Lee 2009). Furthermore, ITC activity is also influenced by their chemical-physical properties, as hydrophobicity and volatility and is specie-specific. (Kim and Lee 2009; Leoni et al. 1997; Nowicki et al. 2019). For instance, sulforaphane, the ITC deriving from the glucoraphanin GSL, particularly concentrated in broccoli and black kale seeds, showed its antimicrobial activity against *Helicobacter pylori* (Fahey et al. 2013); benzyl isothiocyanate was found to be the most effective, among several ITCs tested, against *Campylobacter jejuni*, *Escherichia coli*, and *Staphylococcus aureus*, including some antibiotic-resistant phenotypes (Aires et al. 2009; Dufour et al. 2012; Nowicki et al. 2016; Sofrata et al. 2011); *Moringa peregrine* aqueous extracts were successful tested for the inhibitory activity against resistant strain of *Salmonella enterica* (Saleh et al. 2017)*.* More recently, broths of lactic acid bacteria fermented with *Eruca sativa* seed extracts showed promising results in preventing *Escherichia coli*-induced intestinal inflammation and barrier dysfunction (Bonvicini et al. 2020). In the present work, DSMs containing GSLs belonging to the different classes according to the aglycone R-side chain chemical structure (Table 1) were chosen: sinigrin [allylGSL] (SIN), an aliphatic GSL, glucocheirolin [3-(methylsulfonyl)propyGSL] (GCH) and glucoerucin [4-(methylthio)butylGSL] (GER), two thiofunctionalized GSL with a different sulfur oxidation state, as well as two aromatic GSL, gluconasturtiin [2-phenylethylGSL] (GST), and glucotropaeolin [benzylGSL] (GTL). The different GSL chemical structure determines the chemical and physical properties and biological activity of deriving hydrolysis products (ITCs), where the R-side chain is maintained (Dufour et al. 2015; Leoni et al. 1997). For instance, allylITC (AITC) derived from SIN is a short-chain aliphatic compound that is known for its broad-spectrum biological activity. SIN is exploited in the so-called biofumigation technique owing to its high vapor pressure (Lazzeri et al. 2013). On the other hand, cheirolin [3-(methylsulfonyl)propylITC] (CH), the ITC deriving from GHC, is a little known, non-volatile substance characterized by a slight water solubility, higher than most ITCs (VanEtten and Tookey 2018; Vaughn and Berhow 2005). Erucin [4-(methylthio)butylITC], the ITC produced from GER, is a structural analogue of sulforaphane [(methylsulfinyl)butylITC] (SFN), the most frequently studied ITC for its preventive activity against a variety of cancers, cardiovascular, neurodegenerative diseases, and diabetes (Yang et al. 2016). SFN also showed inhibitory activity against bacteria and fungi in in vitro trials (Johansson et al. 2008). Aromatic ITCs, 2-phenylethylITC (PEITC) and benzylITC (BITC), deriving from GST and GTL respectively, are characterized by low volatility and high hydrophobicity and seem to have high biological activity against some bacterial strains according to literature (Dias et al. 2012; Kim and Lee 2009).

**Table 1:**
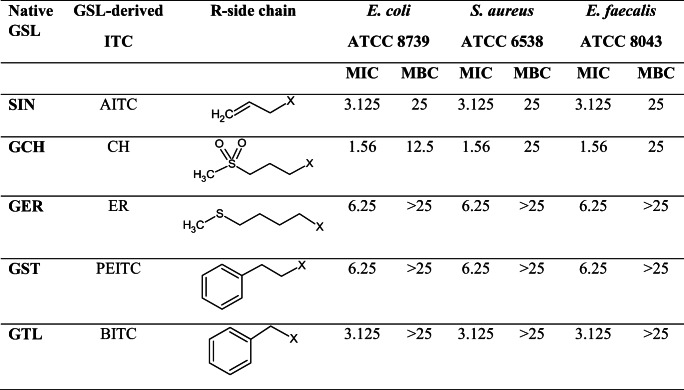
MIC and MBC (mM) values of isotiocyanates (ITCs) produced by glucosinolate (GSL) against the three bacterial pathogens tested. GSLs and respective ITCs are sinigrin (SIN) and allyl-isothiocyanate (AITC); glucocheirolin (GCH) and cheirolin (CH); glucoerucin (GER) and erucin (ER); gluconasturtiin (GST) and phenylethyl-isothiocyanate (PEITC); glucotropaeolin (GTL) and benzyl-isothiocyanate (BITC); GSL or ITC common variable side chain (R) chemical structure is also indicated, where X represents the GSL S-glucopyranosyl thiohydroximate moiety or the ITC (S=C=N-) moiety

GSLs accumulate in seeds during ripening and Brassicaceae defatted seed meal (DSM), a low cost by-product of seed oil extraction that represents a natural bioactive material for biofumigation in soil pathogen containment (Curto et al. 2016). In addition, Brassicaceae DSM is a source of nitrogen (5–6%) and organic matter, with a C:N ratio around 8:1, and for this, it is considered a good soil fertilizer in agricultural production systems, able to better increase nitrogen availability respect to green manure and crop residues (Marchetti et al. 2015; Snyder et al. 2009). At the same time, mustard seed meal soil amendment showed to initially delay C mineralization as compared to flax seed meal (Wang et al. 2012). In this study, the involvement of microbial nitrification inhibition by the GSL containing meal was suggested.

Up to date, no information is available on the activity of Brassicaceae products towards pathogens in complex matrices playing an essential role in pathogenic bacteria diffusion, such as livestock manure.

In the present study, the antibacterial activity of GSL deriving products and related DSMs was studied for its potential to reduce pathogen population in pig manure. The aim of the research was the improvement of pig manure safety by DSM addition, for its potential use as a fertilizer at reduced biological risk in agricultural applications. The pig manure DSM treatment is proposed as an agronomically and economically viable management practice for sustainable crop production able to reduce environmental pollution and human health risk.

In particular, the following have been studied: (i) in vitro activity of products derived from in situ hydrolysis of different pure GSL towards pathogenic strains as *Escherichia coli*, *Staphylococcus aureus*, and *Enterococcus faecalis*; (ii) DSM release kinetics of GSL hydrolysis products in buffer aqueous solutions and in pig manure, under different assay conditions; (iii) in vitro DSM inhibitory effect against the pathogenic strains; (iv) DSM inhibitory effect on pig manure bacterial population.

## Materials and methods

### Glucosinolate and myrosinase

The GSLs were isolated and purified as K^+^ salts from different Brassicaceae seeds according to (Citi et al. 2019). The following GSLs were considered: SIN, 90% w/w, isolated from *Brassica nigra* (L.) W.D.J. Koch; GCH, 93.3% w/w, isolated from *Rapistrum rugosum* (L.) All.; GER, 100% w/w, isolated from *Eruca sativa* Mill.; GST, 98% w/w, isolated from *Barbarea Verna* (Mill.) Asch.; GTL, 100% w/w, isolated from *Lepidium sativum* L. Epiprogoitrin [(2*S*)-2-hydroxybut-3-enylGSL] (EPI), used as an internal standard for GSL analysis, was isolated from *Crambe abyssinica* Hochs t. ex R. E. Fries.

MYR (β-thioglucoside glucohydrolase, EC 3.2.1.147) was purified from *Sinapis alba* seeds as reported in (Pessina et al. 1990). The enzyme (46 U/mL stock solution) was stored at 4 °C in sterile distilled H_2_O until use. MYR activity, expressed in unit (U), was defined as the amount of enzyme able to hydrolyze 1 μmol of SIN/min at pH 6.5 and 37 °C. For in situ hydrolysis of GSLs, 1 U/mL was used (Haack et al. 2010).

### Bacterial strains and growth conditions

Three bacterial strains, namely *Escherichia coli* ATCC 8739, *Staphylococcus aureus* ATCC 6538, and *Enterococcus faecalis* ATCC 8043, were used for antibacterial tests. The cultivation/assay medium was Müeller Hinton cation supplemented Broth (MHB) or Müeller Hinton Agar (MHA) (Becton Dickinson and Company, Cockeysville, MD, USA). Bacterial cultures for antimicrobial testing were prepared by picking one colony from 24-h-old MHA plates and suspending them in MHB (10 mL). Cultures were grown aerobically for 20 h, with continuously shake at 100 rpm at 37 °C. For antibacterial activity assays, cells from each culture were first centrifuged (6000×*g* for 15 min). Cell pellets were washed with sterile phosphate buffered saline solution (PBS) and then diluted to obtain an absorbance value of 0.5 at a wavelength of 600 nm. This suspension was further diluted 1:100 in PBS to obtain an inoculum with a final concentration of 10^5^ CFU/mL.

### Defatted seed meals

*R. rugosum*, *E. sativa*, *B. Verna*, and *L. sativum* seeds were available in the seed collection of Brassicaceae of CREA-CI (Bologna) (Lazzeri et al. 2013). Crops were cultivated in northern Italy, at the experimental station of CREA-CI located at Budrio (Bologna), in the Po Valley area (44° 32’ 00” N; 11° 29’ 33” E, altitude 28 m a.s.l.). Seeds were sown in October 2018–2019, in 90-m^2^ experimental plots at open field level adopting low input techniques for both energy and fertilizers, while no pesticides were applied. The area was characterized by flat land with alluvial deep loamy soil. In June, once the full maturity stage was reached, plants were harvested and threshed by a fixed machine, using sieves suitable for small seeds. After harvesting, seeds were accurately cleaned with a fixed Small-Scale Threshing Equipment (Cicoria Srl, Italy), air-dried to reduce the high residual moisture content, and stored in a dry and dark place at room temperature (RT). Before experiment setup, seeds were finally ground in an ultra-centrifugal mill ZM200 (Retsch GMBH, Germany), sieved at 0.5 mm, and defatted using hexane (1:10 w/v) under agitation, overnight at 21 ± 1 ^∘^C. Before defatting, *R. rugosum* seeds were scarified with a grinder (Bühler-Miag, MLI-204) to remove the very corky no-GSL containing silique.

*B. nigra* seeds were purchased by Nutrien Italia S.p.A (Livorno, Italy), defatted by mechanical extraction (Lazzeri et al. 2010) and further extracted by hexane as described above.

The obtained defatted seed meals (DSMs) were appropriately formulated by a patented procedure able to modulate AITC release in time after watering (Lazzeri et al. 2010). The preparation details must be considered as commercially confidential and their property is of Nutrien Italia S.p.A.

DSMs were characterized for moisture, nitrogen, residual oil, and total phenolic content according to Pagnotta et al. (2019). Dry matter content was determined by oven-drying the seeds at 105 °C for 12 h and evaluating the difference in weight before and after treatment.

The oil content was determined by the standard automated continuous extraction, following the Twisselmann principle, by using a E-816 ECE (Economic Continuous Extraction) extraction unit (BÜCHI Labortechnik AG, Switzerland), and hexane as a solvent. Nitrogen content was determined by the elemental analyzer (LECO Corp., mod. CHN TruSpec, St. Joseph, MI, USA).

Phenolic compounds were extracted with 80% methanol in water, with a 1:20 (w/v) ratio by using ultrasound-assisted extraction (25 min) and determined by the Folin-Ciocalteu method (Dewanto et al. 2002). Gallic acid was used as a standard and the results were expressed as mg gallic acid equivalent (GAE)/g meal.

Total GSL content and profiles were determined by HPLC analysis of desulfo-GSLs following the ISO 9167-1 method (ISO 9167-1:1992/Amd 1:2013), with some minor modifications (Pagnotta et al. 2017), by using a Hewlett-Packard 1100 HPLC equipped with a diode array detector and an Inertsil 5 ODS-3 column (250 × 3 mm). The desulfo-GSLs were detected monitoring their absorbance at 229 nm and identified by UV spectra and HPLC retention times according to a purified standard library. Their amount was estimated trough the internal standard method (by using SIN as internal standard) and published response factors (Wathelet et al. 2004). For *B. nigra* DSM containing SIN, EPI was used as an internal standard, taking into account the experimentally determined response factor for SIN of 0.92. Internal standard SIN and EPI purity was 98.7 and 92%, respectively, as indicated by HPLC-UV chromatograms, and > 96% for both GSLs on a weight basis.

### Antibacterial activity of pure GSL derived hydrolysis products towards pathogenic bacteria

Inhibitory activity against bacterial isolates performed by testing GSL hydrolysis products, derived from in situ hydrolysis of pure native GSL, was assessed in in vitro assays. GSL concentration range of testing was chosen on the basis of ITC relasing trial results.

The minimum inhibitory concentration (MIC) and minimal bactericidal concentration (MBC) methods were used to measure the in vitro activity of each selected GSL hydrolysis product. The MIC is defined as the lowest concentration that inhibits visible microbial growth, while the MBC is the lowest concentration resulting in the death of ≥ 99.9% of the initial inoculum.

Susceptibility testing of strains to GSL hydrolysis product was performed according to ISO (EUCAST 2017; ISO 20776-2 2007; ISO 20776-1 2006) International Guidelines methods for broth dilution susceptibility testing of bacteria and fungi. Bacterial sensitivities were tested using Muller-Hinton medium broth (MHB) (Becton Dickinson and Company, Cockeysville, MD, USA).

For MIC determination, a 50-mM stock solution was first prepared in 2xMHB, for each GSL to be tested and successively filtered (0.2 μm Millipore).

Each stock solution (50 mM) was further serially two-fold diluted (1:2) in 2-mL sterile microcentrifuge tubes (Sarstedt, Germany) in MH broth.

After diluting, GSL in situ hydrolysis was performed by adding 15.4 μL of MYR to each microcentrifuge tube (containing 700 μl of dilutions) before incubating at 37 °C for 30 min. Then, to test GSL hydrolysis product effectiveness, aliquots of 50 μL of each dilution were added to 50 μL of bacterial suspension made in sterile PBS. One hundred μL was dispensed in 96-well microtiter plates (Sarsted, Germany). Beforehand, appropriate control tests, without MYR addition, were performed to evaluate the antimicrobial activity of non-hydrolysed GSLs.

The final concentrations of GSLs tested were 25, 12.5, 6.25, 3.125, 1.56, 0.78, 0.39, and 0.19 mM.

Each microplate was sealed with sterile aluminum foil (SARSTEDT AG & Co. KG, Germany) to avoid leakage, contamination, as well as evaporation, of diluted GSL or GSL hydrolysis product solutions during the assays. Microplates were incubated in aerobiosis at 37 °C for 24 h. Microbial growth was evaluated by optical density (OD_600nm_) through the Multiskan EX spectrophotometer (Thermo Fisher Scientific, Waltham, MA, USA).

Finally, for MBC determination, 10 μL of broth was taken from the well showing no microbial growth, inoculated on MHA plates and incubated at 37 °C in aerobiosis; after the specific incubation time required for each strain, CFU/mL were counted to assess cell viability.

### DSM isothiocyanate release kinetic experiments

#### GSL hydrolysis product release from DSMs under controlled conditions

DSMs were separately incubated at different times in 50 mM potassium phosphate buffer, pH 6.5 and pH 7.5, 1:10 w/v, under agitation at RT in closed vials. The optimum DSM to aqueous solution ratio of 1:10 w/v, which permitted the maximum GSL hydrolysis efficency, was established in preliminary trials (results not shown). The yield of formed hydrolysis products from DSM GSLs, actually ITCs, was indicated as a percentage of DSM initial content of GSL, and determined at 5 min to evaluate the prompt ITC release, and then at 60 min and 24 h to evaluate ITC stability in solutions at longer incubation times. Hydrolysis products were extracted with ethyl acetate according to (Leoni et al. 2004) with some modifications. Two slightly different procedures depending on the different DSM behaviors were applied and their description follows.

*R. rugosum* DSM buffer suspension was centrifuged (30500×*g* for 2 min at 20 °C) after incubation and 1 mL of the collected aqueous phase was extracted with 2 mL of ethyl acetate for 30 min, under mild orbital shaking. A more vigorous agitation did not allow to recover ITC because of the formation of a thick gel during incubation, probably as a consequence of mucilage solubilization from DSM (North et al. 2014). Finally, the organic phase was recovered by centrifugation and analyzed by gas-chromatography.

Instead, buffer suspensions with the other DSMs were directly extracted with 2 mL of ethyl acetate, without previous centrifugation and solid phase separation to avoid a great loss in ITCs recovery. The mixture was subjected to vigorous agitation by vortex for at least 5 min and finally centrifuged (30500x*g* for 20 min at 20 °C). The organic phase was recovered and analyzed by gas-chromatography.

#### Release of GSL hydrolysis products from DSMs in pig manure

DSMs were singly incubated in a liquid pig manure consisting of a mixture of pig waste and wastewater used to remove the waste from pens. Slurry sample from swine barn containing weaning piglets was collected from approximately 5–10 cm below the surface of pits and processed within 4 h.

The same procedure of DSM extraction and analysis described above (1:10 w/v, under agitation at RT) was followed. The experiments were conducted both in closed and open vials, the latter simulating the treatment in the sewage storage tank.

#### ITC gas chromatographic analysis

Extracted ITCs in ethyl acetate solution were injected in a Varian Saturn CP-3800 gas chromatograph (GC) coupled with a flame ionization detector (FID), equipped with a J&W HP-5 column (Agilent technologies, 30 m × 0.25 mm i.d., 0.25 μm film thickness). The analytical conditions were: injection temperature 250 °C; detector temperature 240 °C; flow of N_2_ as a carrier gas 1 mL/min; column oven temperature initially maintained at 60 °C for 4 min, followed by a gradient to 220 °C, with a rate of 10 °C/min, and finally held for 2 min. The split mode (1:20) was used for injection. ITCs were identified by comparison of the compound retention times with pure ITC standards analyzed under the same conditions. ITCs deriving from *B. nigra*, *R.* r*ugosum*, *E. sativa*, and *B. verna* were quantified by the addition of the internal standard BITC to the extractant ethyl acetate (3.8 mM) and the use of gas-chromatographic response coefficients for calculation. Conversely, PEITC was used as an internal standard for BITC quantification in *L. sativum*. Response coefficients were calculated by the ratio of the slope of the calibration curves obtained from the studied ITC in pure form and the internal standard solutions, in the range of 0.3–10 mM. Pure standards of AITC, PEITC, and BITC were purchased from Sigma-Aldrich, while CH and erucin [4-(methylthio)butylITC] (ER) were produced and purified from the precursor GSLs, GCH and GER, as described in Citi et al. (2019), with a purity > 98% (w/w). Recovery of ITC derived from pure GSL hydrolysis by MYR and ethyl acetate extraction was between 85 and 90%, as reported by (Leoni et al. 2004). Recovery precision, determined as a relative standard deviation, was 4.2 and 6.4% for pure standard BITC and PEITC, respectively.

### Antibacterial activity of DSMs towards pathogenic bacteria

The plate count method was used to measure in vitro the activity of each selected DSM towards test bacterial strains.

For antibacterial assays, 200 mg of DSM was weighed in a sterile 50-mL tube and dissolved in 1 mL 2xMHB. Bacterial suspensions (1 mL each), diluted with PBS to obtain an inoculum with a final concentration of 10^5^ CFU/mL, were added to 2xMHB and the tubes were incubated at 37 °C for 24 h in aerobiosis. After incubation, for enumerating viable bacterial cells, suspensions were tenfold diluted to obtain a cellular concentration in sterile PBS from 10^-1^ up to 10^-9^ CFU/mL. Aliquots of 1 mL of each suspension were then plated onto MHA in triplicate. Plates were then incubated at 37 °C. Colony enumeration was carried out after 24 h and results were expressed as CFU/mL.

### Antibacterial activity of DSMs towards pathogenic bacteria of pig manure

The antimicrobial activity of *R. rugosum* and *B. nigra* DSMs was assayed towards pig liquid manure bacteria by means of plate count method.

To enumerate each selected bacterial parameter, 2 g of DMS was weighed in a sterile 50-mL tube and mixed with 18 g of pig slurry (1:10 w/v). Two mL of sterile PBS (pH 7.2) was mixed with 18 g of pig slurry and used as a control. Both the suspensions were incubated at RT for 24 h. Before and after incubation, suspensions were serially tenfold diluted in sterile PBS from 10^-1^ up to 10^-9^ CFU/mL. Aliquots of 1 mL of each dilution were then plated onto respective medium in triplicate. Plates were then incubated at different temperature depending on the microorganism searched, and colony enumeration was carried out after 24 or 48 h of incubation, as described in the following. Plate counts of total coliforms and *E. coli* (CFU/mL) were determined on Chromocult coliformen agar (Merck) with incubation at 37 °C for 24 h. Plate counts of faecal enterococci (CFU/mL) were determined on Slanetz and Bartley Agar (Merck, Darmstadt, Germany) with incubation at 37 °C for 48 h.

Plates counts of total aerobic and anaerobic mesophilic bacteria were determined on LB agar (Merck, Darmstadt, Germany) with incubation at both 37 and 22 °C. The anaerobic atmosphere was obtained using the GasPak EZ Anaerobic Pouch system (BD). The presence of amoxicillin resistant aerobic and anaerobic bacteria was also determined by plating on LB agar containing 100 mg/L of amoxicillin (Sigma Aldrich).

### Statistical analysis

**Fig. 1:**
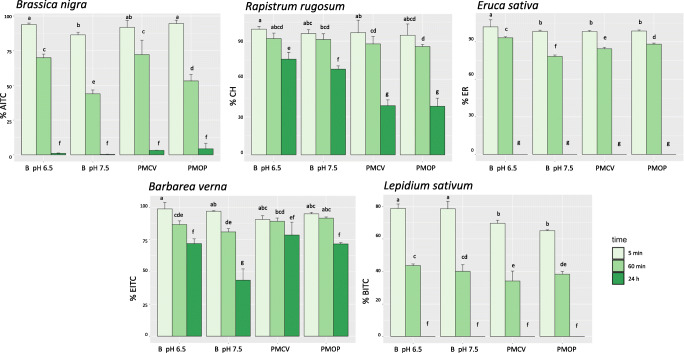
Isothiocyanates release in buffer (B), at pH 6.5 or 7.5, and in pig manure in closed or open vials (PMCV or PMOP, respectively), from defatted seed meals. Isothiocyanate yields are expressed as a percentage of defatted seed meal glucosinolate initial content. AITC, allyl-isothiocyanate; CH, cheirolin; ER, erucin; PEITC, phenylethyl-isothiocyanate; BITC, benzyl-isothiocyanate. Mean values (n > 3) followed by different letters are significantly different according to the LSD test (P < 0.05)

**Fig. 2:**
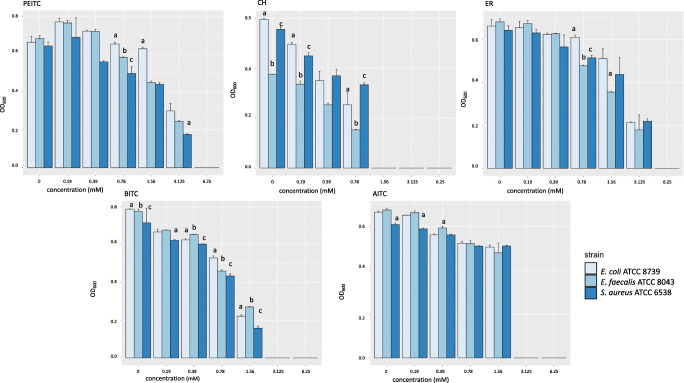
Effect of isothiocyanate solutions on the growth of *E. coli*, *S. aureus*, and *E. faecalis* strains*.* Tested isothiocyanate are allyl-isothiocyanate (AITC); cheirolin (CH); erucin (ER); phenylethyl-isothiocyanate (PEITC); and benzyl-isothiocyanate (BITC). On X axis, initial glucosinolate concentration in mM, and on Y axis, optical density (OD) of bacterial growth at 600 nm. Statistically significant differences among inhibitory effect towards the pathogens are evidenced by letters

**Fig. 3:**
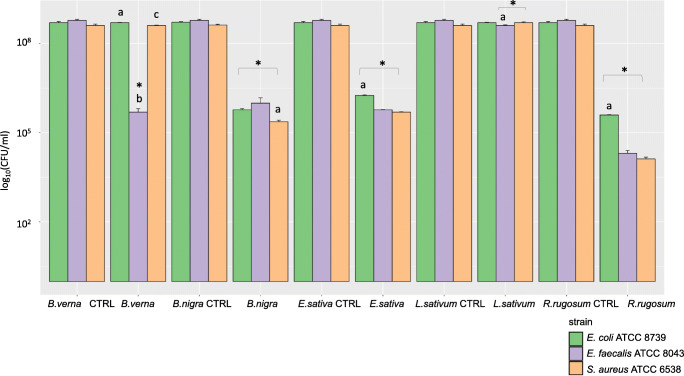
Antibacterial activity of brassicas defatted seed meals towards *E. coli*, *S. aureus*, and *E. faecalis* strains. CTRL stands for the control for each brassicas defatted seed meal. Statistically significant differences among inhibitory effect towards the pathogens are evidenced by letters. Statistically significant differences between DSM effect on each pathogen and the control are evidenced by asterisks

All experiments were expressed as a mean of at least three independent experiments. The statistical analysis of the results was performed with R 3.4.4. 2018 (agricolae package) software (Foundation for Statistical Computing, Vienna, Austria) (McMurdie and Holmes 2013). ITC release kinetic experiment data (Fig. 1) were subjected to a one-way analysis of variance employing the least significant difference (LSD) test to assess significant differences between the samples analyzed (P < 0.05). MIC and MBC determination and antibacterial experiment results were subjected to ANOVA and Tukey test analysis and statistically significant differences were evaluated by one-way (Fig. 2, Fig. 3, and Table 2) and two-way ANOVA (Supplementary Table S1 and Table S2). Results were considered significant at P ≤ 0.05.

**Table 2:** Growth of selected bacterial groups at 0 (T0) and 24 h (T24) in the absence or presence of *R. rugosum* DSM (A) or *B. nigra* DSM (B), in aerobiosis and anaerobiosis and at 22 and 37 °C. Statistically significant differences (*P* value <0.005) are calculated on the growth decrease (Δ). The values present the average ± standard deviation of growth decrease. The statistically significant differences are evidenced in *P* value column

A		*R. rugosum* 0 mg/mL			*R. rugosum* 100 mg/mL			
		T0	T24	Δ	T0	T24	Δ	*P* value
22°C	AEROBIC	1,4×10^5^ ± 10^4^	1,03×10^5^ ± 5,77×10^3^	3,67×10^4^±1,15×10^4^	1,43×10^5^ ± 5,77×10^3^	2,27×10^4^ ± 5,77×10^3^	1,21×10^5^	0.00034
	ANAEROBIC	1,4×10^5^ ± 10^4^	1,33×10^5^ ± 1,53×10^4^	6,67×10^4^±1,15×10^4^	1,47×10^5^ ± 5,77×10^3^	1,03×10^5^ ± 5,77×10^3^	4,33×10^4^	0.0079
37°C	AEROBIC	1,9 ×10^5^ ± 10^4^	1,6×10^5^ ± 5,77×10^3^	2,33×10^4^±1,53×10^4^	1,6 ×10^5^ ± ×10^4^	10^5^ ± 10^4^	6 ×10^4^	0.065
	ANAEROBIC	4 ×10^5^ ± 10^4^	2,9×10^5^ ± 10^4^	1,10 ×10^5^±1,7310^4^	4,77×10^5^ ± 2,52×10^4^	1,4×10^5^ ± 10^4^	3,37×10^5^	7,68×10^-5^
22°C	AEROBIC+amox	1,4 ×10^5^ ± 10^4^	1,3×10^5^ ± 10^4^	10^4^±2×10^4^	1,28×10^5^ ± 5,86×10^3^	2,4×10^4^± 10^4^	1,04 ×10^5^	0.00149
	ANAEROBIC+amox	3,1 ×10^5^ ± 10^4^	2,3×10^5^ ± 10^4^	3,67×10^4^±2×10^4^	3,2×10^5^ ± 10^4^	3,3×10^4^ ± 5,77×10^2^	1,57 ×10^5^	1,85×10^-5^
37°C	AEROBIC+amox	1,4 ×10^5^ ± 10^4^	1,36×10^5^ ± 5,77×10^3^	3,33×10^3^±1,53×10^4^	1,2 ×10^5^ ± 10^4^	2,9×10^4^± 10^3^	9,10 ×10^4^	0.00102
	ANAEROBIC+amo	2,5 ×10^5^ ± 10^4^	2,1×10^5^ ± 5,77×10^3^	3,67×10^4^±1,53×10^4^	2,6 ×10^5^ ± 10^4^	1,03×10^5^ ± 10^3^	1,57×10^5^	0.00041
	TOTAL COLIFORMS	600 ± 20	560 ± 10	40±17,3	610 ± 10	5,03×10^2^± 5,77	1,07×10^2^±5,77	0.0032
	*Escherichia coli*	2,4 ×10^3^ ± 10^2^	2×10^3^ ± 10^2^	4×10^4^±10^2^	2,2×10^3^± 10^2^	0	2,2×10^3^±10^2^	3,17×10^-5^
	ENTEROCOCCI	2,3×10^4^ ± 10^3^	2,2×10^4^± 10^3^	10^3^±2×10^3^	2,4×10^4^ ± 10^3^	2,1×10^4^± 10^3^	3×10^3^±1,73×10^3^	0.26
B		*B. nigra* 0 mg/mL			*B. nigra* 100 mg/mL			
		T0	T24	Δ	T0	T24	Δ	*P* value
22°C	AEROBIC	1,43×10^4^ ± 5,77×10^2^	1,27×10^4^ ± 5,77×10^2^	1,67×10^3^±1,15×10^3^	2,3×10^4^ ± 10^2^	1,4 ×10^4^ ± 10^2^	9×10^3^ ±100	0.00039
	ANAEROBIC	1,97×10^5^ ± 5,77×10^3^	10^4^± 10^4^	1,67×10^4^±1,15×10^4^	6×10^4^ ± 10^3^	1,47×10^4^ ± 7,07×10^2^	4,53×10^4^±1,15×10^3^	0.0128
37°C	AEROBIC	3,07 ×10^4^± 1,53×10^3^	2,87×10^4^ ± 5,77×10^2^	2×10^3^±1,73×10^3^	3,07 ×10^5^± 1,53×10^3^	2×10^4^ ± 10^3^	1,07 ×10^4^±1,15×10^3^	0.0028
	ANAEROBIC	1,9×10^5^ ± 10^4^	1,87×10^5^± 1,53×10^4^	3,33×10^3^±2,31×10^4^	1,6×10^5^ ± 10^4^	5×10^4^ ± 10^3^	1,1×10^5^±9×10^3^	0.0017
22°C	AEROBIC+amox	9,3×10^4^ ± 5,77×10^2^	8,67×10^3^ ± 5,77×10^2^	6,67 ×10^2^±1,15×10^3^	8×10^3^±10^2^	2,7×10^2^ ± 10	7,73×10^3^ ±90	0.00045
	ANAEROBIC+amox	2,9×10^5^± 5,77×10^2^	2,7×10^4^ ± 10^4^	2,33×10^3^ ±1,15×10^3^	2,8×10^4^ ± 10^3^	1,6×10^3^ ± 10^2^	2,64×10^4^ ±1,05 ×10^3^	2,96×10^-5^
37°C	AEROBIC+amox	2,5×10^4^ ± 5×10^2^	2,07×10^4^± 1,53×10^3^	4,33×10^3^ ±1,26×10^3^	2,5×10^4^± 10^3^	10^4^ ± 10^3^	7,73 10^3^±9	0.00144
	ANAEROBIC+amox	1,2×10^5^± 5,77×10^3^	1,2×10^5^± 10^4^	3,33×10^3^ ±5,77×10^3^	1,3×10^5^ ± 10^4^	4,5×10^4^ ± 10^3^	8,5010^4^±1,1 ×10^4^	0.00034
	TOTAL COLIFORMS	3,2×10^3^ ± 10^2^	2,97×10^3^ ± 5,7×10	2,33×10^2^±1,53×10^2^	3×10^3^ ± 10^2^	2,87×10^3^ ± 1,15×10^2^	1,33×10^2^±1,53×10^2^	0.467
	*Escherichia coli*	9,8×10^2^ ± 10	9×10^2^± 10	80 ±0	9,76×10^2^ ± 5,3	8,9×10^2^ ± 5,77	79,3 ±1,15	0.926
	ENTEROCOCCI	7×10^3^ ± 10^2^	6,7×10^3^ ± 10^2^	3 ×10^2^ ±10^2^	7,03×10^3^± 5,7×10	6,63×10^3^ ± 1,53×10^2^	4×10^2^±10^2^	0.287

## Results and discussion

### GSL hydrolysis product antibacterial activity towards pathogenic bacteria

The inhibition activity of hydrolysis product formed by in situ hydrolysis of GSL in pure form was investigated in in vitro assays, towards some pathogenic bacteria.

ITCs were produced in situ by adding purified enzyme MYR in MHB where pure GSL were dissolved. The GSL hydrolysis efficiency was preliminary verified by ethyl acetate extraction of the incubated solution followed by GC-FID analysis. ITCs were identified as the main products (> 95%) that were quantitatively formed from the corresponding GSLs, as previously reported (Leoni et al. 2004). The antibacterial activity of the formed ITCs was thus evaluated at different starting GSL concentrations (from 25 to 0.19 mM). The MIC values were assessed by broth microdilution technique towards three human putative pathogens (namely, *E. coli*, *S. aureus*, and *E. faecalis*) and the initial GSL concentration and the stoichiometry of the reaction were taken into account.

The MICs of the five ITCs that were tested against three bacterial strains are showed in Table 1. For each ITC solution tested, a different antibacterial effect depending on the strain used (Supplementary Table S1) was observed. In particular, considering the same concentration for the different ITC solutions, we found statistically significant differences in all three bacterial strains (Supplementary Table S1). Interestingly, CH showed the lowest MIC at 1.56 mM, which corresponded to the highest antibacterial activity, followed by AITC and BITC, both at 3.125 mM. Both ER and PEITC showed the highest MIC value at 6.125 mM (Fig. 2).

The lowest dose tested (0.19 mM) for CH showed a statistically significant effect in the reduction of growth in *E.coli* and *S.aureus* strains (Supplementary Table S2). Rising from the concentration of 0.78 mM, all ITC solutions impact significantly on the growth of the strains tested (Supplementary Table S2). Globally, ER and BITC seemed to be the less efficient ITC in the decrease of strains growth, considering the lowest concentrations tested (0.19 and 0.39 mM, respectively).

The MBC of the ITC solutions resulted almost eight times higher than corresponding MIC. CH solution showed a MIC of 1.56 mM and a MBC of 12.5 mM for *E. coli*, *S. aureus*, and *E. faecalis*. AITC solution showed a MIC of 3.125 mM and a MBC of 25 mM towards all the three target microorganisms, while BITC, PEITC, and ER solutions showed a MIC in the range 3.125–6.25 mM with a MBC > 25 mM for all of them (Table 1).

### Brassicaceae DSM characterization

Brassicaceae seeds were selected according to the qualitative and quantitative GSL content, as well as the agronomic features of source crops (Lazzeri et al. 2004, 2013; Mohamed et al. 2020). In this context, all the chosen crops showed good agronomic performances, such as low input requirements, high robustness and adaptability, with the exception of *R. rugosum* which, as an invasive weed plant, presented a more difficult management (Mobli et al. 2020). Anyway, this plant was chosen for the particular GSL seed content, whose biological activity has been little investigated and therefore deserved particular attention.

DSMs were obtained after oil extraction in mild condition, to preserve bioactive compounds from degradation. They were finally formulated (Lazzeri et al. 2010) in order to standardize their MYR activity content. Formulated DSM chemical characterization results are shown in Table 3. DSMs containing one prevalent GSL of those tested in preliminar in vitro assay (Table 3) were selected. Indeed, *B. nigra* mainly contained SIN, *R. rugosum* and *E. sativa* contained GCH and GER, respectively, while *B. verna* and *L. sativum* contained the two aromatic GSL, GST and GTL, respectively. *E. sativa* also contained 10.7 ± 0.4 μmol/g of glucoraphanin ((*R*S)-4-(methylsulfinyl)butyl GSL) (not showed). The DSM total phenolic content (TPC) was also quantified, as phenolics could have a role in the antibacterial activity, beside their known antioxidant activity (Bouarab-Chibane et al. 2019). Their values ranged between 7.1 mg GAE/g in *R. rugsosum* and around 12 mg GAE/g in both *L. sativum* and *B. verna* DSMs (Table 3) and were in line with the TPC content of other DSMs from plants of the same family (Borș et al. 2017; Lucarini et al. 2019; Mohamed et al. 2016; Pagnotta et al. 2019). Regarding the other DSM parameters determined, the residual oil content was low, as expected from the hexane defatting procedure, and less than 11% for all the DSMs considered (Table 3). The antibacterial activity of brassica seed oil components, in the form of concentrated essential oils, or solvent extracts rich in phytochemicals as flavonoids, alkaloids, fatty acids, has been already assessed (Alqahtani et al. 2019; Khoobchandani et al. 2010; Obi et al. 2009). In the present work, the role of the oil component could be considered negligible owing to the poor residual oil content of DSMs (≤ 10.4% of dry matter, Table 3) and to the bioassay conditions in which DSMs activity have being tested (i.e., aqueous media). Finally, the nitrogen content was higher for *B. nigra* and *R. rugosum* (Table 3). The good DSM nitrogen content could be eventually exploited in agricultural applications for soil fertilization and this aspect should deserve more attention in further studies (Lazzeri et al. 2013; Marchetti et al. 2015).

### GSL hydrolysis product release from DSMs in buffer and pig manure

The release of GSL hydrolysis products from DSMs is a simultaneous two-step process involving the GSL extraction from the DSM into the medium and the GSL hydrolysis catalyzed by MYR, in the presence of water. This releasing process was first investigated under controlled conditions by incubating, in closed vials, the DSM in buffer at two different pH values. In particular, DSM was incubated at pH 6.5 in order to obtain the maximum yield of ITC formation to the detriment of other hydrolysis products, according to literature (Bones and Rossiter 2006). In addition, the same trials were conducted at pH 7.5, which was the pH of the pig manure investigated in this study. The manure was selected because its pH represented the average pH of pig manures previously tested. Finally, the buffer was substituted by the pig manure and experiments were conducted in both closed and open vials under the same assay conditions. Results obtained from different DSMs are shown in Fig. 1.

ITCs were the main products, quantitatively formed from the respective GSLs extracted from the DSMs, in buffer at pH 6.5 or 7.5 and in pig manure too, while no other hydrolysis products were reavealed by GC-FID analysis of the extracts. The maximum GSL extraction and ITC hydrolysis yield, calculated as a percentage of the initial DSM GSL content, was > 90% for both *B. nigra* and *B. verna* and > 95% for both *R. rugosum* and *E. sativa*, and it was obtained soon after 5 min of incubation of all the DSMs, regardless of the medium and incubation conditions. An exception was represented by *L. sativum* from which lower yields (≤ 80%) were obtained (see below).

The good ITC releasing efficiency was confirmed by the analysis performed on residual DSMs that were collected after incubation. Here, the GSL content was less than 10% for all the DSMs. These results demonstrated that pig manure pH and components, as proteins or possible enzyme inhibitors, did not affect the DSM release efficiency, in terms of GSL extraction, GSL conversion into ITC by MYR, and ITC detection. This result was thus a good premise for further investigations. On the other hand, a different behavior concerning the ITC stability and extractability, depending on the medium, was observed among tested DSMs at longer incubation time (Fig. 1). ITCs are in fact very reactive electrophilic compounds that easily react with hydroxyl, amino, or thiol groups of proteins or other compounds, often forming inactive products, thus possibly affecting their detection or biological activity (Zhang 2012).

AITC from *B. nigra* DSM achieved maximum concentration after 5 min of incubation, that was 17.4 and 16.0 mM in buffer at pH 6.5 and 7.5, respectively, and 17.0 mM in pig manure (Fig. 1). Nevertheless, its detection at longer incubation time, as soon as 60 min, revealed significant lower yields. The decrease was faster in both buffer at pH 7.5 and in pig manure in open vials and led to a very low concentration in all the samples after 24 h (from 0.3 to 4.4 mM). AITC is a molecule that is known to be unstable in buffer or aqueous media (Luang-In and Rossiter 2015) and alkaline pH values seem to decrease its stability. Furthermore, it is a volatile molecule, and this characteristic would explain the faster loss in open vials. Anyway, microbial community in pig manure too could have contributed to AITC degradation (Leoni et al. 2004; Liu et al. 2020).

Instead, CH released from *R. rugosum*, the aliphatic thiofunctionalized ITC, was found more stable. The maximum concentration after 5 min of incubation was found at 18.2 and 17.5 mM in buffer at pH 6.5 and 7.5, respectively, and 17.7 and 17.3 mM in pig manure in closed and open vial, respectively (Fig. 1). The decrease of concentration after 1 h of incubation was less than 10% for all samples with respect to the values at 5 min. After 24 h, the yield of conversion was 68% in buffer at pH 7.5 and 76% at pH 6.5, and CH concentration was 11.7 and 14.0 mM, respectively. Instead, a higher decrease was observed in pig manure at 24 h, with no significant difference between experiments conducted in closed or open vials, confirming the slight water solubility and low volatility of the molecule (VanEtten and Tookey 2018; Vaughn and Berhow 2005). Anyway, CH concentration in pig manure after 24 h, even if lower than in buffer, was still relevant (7 mM; yields around 40%).

A similar behavior was observed for PEITC released by *B. verna* that is a hydrophobic molecule belonging to the class of the aromatic ITCs. PEITC maximum concentration after 5 min was registered as 12–13 mM and slightly dropped to 9–10 mM after 24 h in all solutions, except for buffer at pH 7.5, where it was found at 5.8 mM (Fig. 1).

*E. sativa* DSM efficiently released the aliphatic thiofunctionalized ER with a maximum concentration in 5 min of 12.6 mM in buffer at pH 6.5 and of 12.2 mM in the other solutions, which was almost maintained after 1 h of incubation (Fig. 1). Nevertheless, at prolonged incubation time, ITC recovery was more difficult because the suspension became thick owing to DSM mucilage swelling (Koocheki et al. 2012). The phenomenon probably caused the failure of ER detection after 24 h in all the samples.

The same problem was encountered with *L. sativum* DSM. The mucilage around the outer layer of seeds of this plant produces highly viscous solution when it swells (Behrouzian et al. 2014). The mucilage could have entrapped the BITC released from the DSM, determining a lower yield, even after 5 min of incubation (from 79% in buffer at pH 6.5 to 65% in pig manure and open vial) while at 24 h, as for ER, no BITC was found in the analyzed extracts (Fig. 1). Similar results with *L. sativum* DSM were obtained by Leoni et al. (2004). Anyway, the residual GSL content of the DSM collected after incubation for different times was < 3%, demonstrating that a complete GSL extraction occurred.

In the end, the ITC concentrations released by DSMs reached the MIC values previously found in in vitro assays in the first 5 min of incubations, and were differently maintained according to the DSM characteristics and ITC stability in buffer and pig manure. On the contrary, all the ITC concentrations were below the previously determined MBC values, except for CH released from *R. rugosum*, apparently the most active ITC, which reached the MBC concentration found against *E. coli.*

### Antimicrobial activity of DSMs towards pathogenic bacteria

Once verified the ITC releasing efficiency of the five considered DSMs, their ability to inhibit or reduce the growth of the three microbial putative pathogens were tested. Each bacteria was enumerated on bacterial suspension maintained in contact with 200 mg of a selected DSM for 24 h at RT. The results of the antimicrobial screening of the five DSMs are given in Table 4 and Fig. 3.

**Table 3:** Chemical characterization of Brassica defatted seed meals (DSMs). Nitrogen (N) and oil contents are expressed as weight percentage on a dry matter basis. Average values ± standard deviation (n = 3) are shown. GSL (glucosinolate): SIN (sinigrin); GCH (glucocheirolin); GER (glucoerucin); GST (gluconasturtiin); GTL (glucotropaeolin). GAE: gallic acid equivalent. TPC (total phenolic content), oil, and N mean values, followed by different letters, are significantly different according to the LSD test (P < 0.05)

DSMs	N %	Oil %	GSL μmol/g		TPC mg GAE/g
*R. rugosum*	7.6 ± 0.1 a	10.4 ± 0.1* a	GCH	182.6 ± 3.3	7.1 ± 0.4 c
*E. sativa*	6.5 ± 0.1 b	9.4 ± 0.3* b	GER	123.7 ± 0.1	9.3 ± 0.3 b
*B. verna*	4.5 ± 0.1 d	6.2 ± 0.3 c	GST	132.5 ± 4.3	12.4 ± 0.1 a
*L. sativum*	5.7 ± 0.1 c	8.9 ± 0.2 b	GTL	172.1 ± 4.5	11.9 ± 0.2 a
*B. nigra*	7.4 ± 0.1 a	2.8 ± 0.1 d	SIN	185.6 ± 1.1	8.9 ± 0.3 b

*data from Matteo et al. 2018

*R. rugosum* DSM resulted the most active among the different DSMs tested, more on *S. aureus* and *E. faecalis* strain respect to *E. coli* (Fig. 3). This result could be attributed both to the intrinsic biological activity of the ITC, as it was verified by using the corresponding pure solution of CH ITC (Table 1), and to its stability as valuated in the DSM releasing assay (Fig. 1). Nevertheless, other DSM components as phenolic compounds could have contributed to the antibacterial activity as well. *B. nigra* and *E. sativa* indeed, showed high activity of inhibition too, despite the less favorable pure biological activity and stability results observed for their corresponding ITCs (AITC and GTL, Table 1). Interestingly, the effect of *B. verna*, whose PEITC showed low antibacterial activity when tested in pure form (Table 1), significantly decreased the growth of *E. faecalis*, while it did not inhibit the other two strains. On the contrary, *L. sativum* DSM showed no antibacterial activity, despite the pure BITC showed good performances (Table 1).

Based on the results of antimicrobial activity relating to both pure GSLs and DSM, the *R. rugosum* DSM containing GCH and *B. nigra* DSM containing SIN were chosen to test their antibacterial activity towards bacteria present in the pig manure.

### Antibacterial activity of DSMs towards bacterial load in pig manure

The antibacterial effect of *R. rugosum* and *B. nigra* DSMs on different groups of bacteria in pig manure (i.e., mesophilic aerobic and anaerobic bacteria, mesophilic aerobic, and anaerobic amoxicillin resistant bacteria, total coliforms, *E. coli* and enterococci) was studied (Table 2). The microbial response to DSM treatment and the population dynamics of the manure microbial community were evaluated measuring the decrease in viable titers after 24 h (T24) with respect to time 0 (T0).

**Table 4:** Antimicrobial activity of the selected defatted seed meals (DSMs) towards bacterial strains. Counts are expressed as log_10_ CFU/mL. Data are expressed as means ± SD for at least three replicates. Glucosinolates (GSLs); SIN: sinigrin; GCH: glucocheirolin; GER: glucoerucin; GST: gluconasturtiin; GTL: glucotropaeolin

DSMs	GSLs	*E. coli* ATCC 8739		*S. aureus* ATCC 6538		*E. faecalis* ATCC 8340	
		0 mg	200 mg	0 mg	200 mg	0 mg	200 mg
*B. nigra*	SIN	8.71 ± 0.02	5.78 ± 0.04	8.62 ± 0.03	5.36 ± 0.06	8.78 ± 0.04	6.00 ± 0.24
*R. rugosum*	GCH	8.70 ± 0.04	5.60 ± 0.01	8.60 ± 0.05	4.11 ± 0.07	8.78 ± 0.04	4.30 ± 0.11
*E. sativa*	GER	8.70 ± 0.04	6.26 ± 0.02	8.60 ± 0.05	5.70 ± 0.01	8.78 ± 0.04	5.78 ± 0.01
*B. verna*	GST	8.70 ± 0.04	8.70 ± 0.01	8.60 ± 0.05	8.60 ± 0.02	8.78 ± 0.04	5.70 ± 0.13
*L. sativum*	GTL	8.70 ± 0.04	8.70 ± 0.01	8.60 ± 0.05	8.70 ± 0.03	8.78 ± 0.04	8.60 ± 0.03

Both *R. rugosum* and *B. nigra* DSMs significantly decreased the growth of the mesophilic aerobic and anaerobic bacteria, as well as mesophilic aerobic and anaerobic amoxicillin-resistant bacteria in every condition tested (P value < 0.05) (Table 2). *R. rugosum* showed a significant inhibitory effect on bacterial growth of total coliforms and *E. coli*, confirming the good antibacterial activity of its DSM, whereas no significant inhibitory effect was observed on enterococci growth (Table 2).

*B. nigra* did not show any effect on total coliforms, *E. coli* and enterococci growth (Table 2). In control samples without the addiction of DSMs, no difference in the bacterial count after 24 h with respect to time zero (T24 and T0, respectively) was observed.

Results demonstrated that the counts of all investigated bacterial groups were differently affected after the addition of selected DSMs.

## Conclusions

Biofumigation is known as an effective agronomic technique, based on the use of Brassicaceae plants and derived products with the intention of enabling ITC-mediated pesticidal effects. This technique is now a sustainable and environmental friendly strategy to manage soil-borne pathogens. In the present work, the use of this technique for bacteria containment in pig slurry was for the first time investigated.

ITCs, that were derived from hydrolysis of pure GSLs, confirmed their antibacterial action. In particular, CH and AITC showed high antimicrobial activity towards *E. coli*, *S. aureus*, and *E. faecalis* in in vitro assays. The efficient and fast ITC release from Brassicaceae DSMs, both in buffer and in pig manure, was verified and the antibacterial activity was confirmed for *R. rugosum* and *B. nigra* DSMs, sources of CH and AITC, respectively, against the three pathogens tested.

Concerning the antibacterial activity of these DSMs in pig manure, after treatment, a bacterial growth reduction of at least one order of magnitude was generally observed among total aerobic, anaerobic, and amoxicillin-resistant bacteria, thus suggesting that the use of DSMs can help in decreasing the bacterial load of pig manure. The reduced pathogenic content of the manure added with DSMs paves the way to produce a fertilizer at a reduced risk potential for crops, animals, and, more general, the environment.

Practical applications of DSMs to animal manure (e.g., through addition into manure tanks in the absence of treatment plants, as well as into the liquid or solid fractions coming from manure solid-liquid separation plants) deserve further investigations, especially regarding the fertilizing effects on soils and crops of the DSM-derived additional nitrogen input to pig manure, usually quite rich of nitrate.

It has to be underlined that an integrated approach which exploits the use of DSM in combination with other possible sanitizing treatments to contain pathogens can be promoted as well.

## Supplementary Information

ESM 1 (DOCX 36 kb)

## Data Availability

All data generated or analyzed during this study are included in this published article. This manuscript has not been previously published, and the paper is currently not under consideration by another journal. All authors have approved and agreed to submit the manuscript to this journal.
