# Supplementary material for: Effect of bioactive compounds released from Brassicaceae defatted seed meals on bacterial load in pig manure
Source: Environ Sci Pollut Res Int. 2021 Jun 30;28(44):62353–67. doi: 10.1007/s11356-021-14321-7 (PMC8589757; doi:10.1007/s11356-021-14321-7)
Supplement: Supplementary file 1 — (DOCX 36 kb) [file 11356_2021_14321_MOESM1_ESM.docx]

**Effect of bioactive compounds released from *Brassicaceae* defatted seed meals on bacterial load in pig manure**

Luisa Ugolini^1^, Donatella Scarafile^2^, Roberto Matteo^1^, Eleonora Pagnotta^1^, Lorena Malaguti^1^, Luca Lazzeri^1^, Monica Modesto^2^*, Alice Checcucci^2^, Paola Mattarelli^2^, Ilaria Braschi^2^

**Supplementary information**

**Table S1.** Interactions between ITC solutions and concentrations tested. P (p < 0.005) and Q values were evaluated with Tuckey post-hoc and two-way ANOVA. *Q=adj p value*

| ***E. coli* ATCC 8739** |  | **Q** | **p** |
| --- | --- | --- | --- |
| CH-3,125 | ER-3,125 | 24,21 | 4,35E-10 |
| CH-3,125 | PEITC-3,125 | 33,93 | 4,35E-10 |
| CH-1,56 | ER-1,56 | 57,69 | 4,35E-10 |
| CH-1,56 | PEITC-1,56 | 70,87 | 4,35E-10 |
| CH-1,56 | BITC-1,56 | 24,97 | 4,35E-10 |
| CH-1,56 | AITC-1,56 | 57,05 | 4,35E-10 |
| CH-0,78 | ER-0,78 | 40,25 | 4,35E-10 |
| CH-0,78 | PEITC-0,78 | 45,11 | 4,35E-10 |
| CH-0,78 | BITC-0,78 | 31,18 | 4,35E-10 |
| CH-0,78 | AITC-0,78 | 30,35 | 4,35E-10 |
| CH-0,19 | ER-0,19 | 18,04 | 4,35E-10 |
| CH-0,19 | PEITC-0,19 | 30,99 | 4,35E-10 |
| CH-0,19 | BITC-0,19 | 19,43 | 4,35E-10 |
| CH-0,19 | AITC-0,19 | 17,58 | 4,35E-10 |
| ER-3,125 | PEITC-3,125 | 9,715 | 7,64E-07 |
| ER-3,125 | BITC-3,125 | 24,21 | 4,35E-10 |
| ER-3,125 | AITC-3,125 | 24,21 | 4,35E-10 |
| ER-1,56 | PEITC-1,56 | 13,18 | 4,43E-10 |
| ER-1,56 | BITC-1,56 | 32,72 | 4,35E-10 |
| ER-0,78 | BITC-0,78 | 9,075 | 5,85E-06 |
| ER-0,78 | AITC-0,78 | 9,903 | 4,16E-07 |
| ER-0,39 | PEITC-0,39 | 10,92 | 1,52E-08 |
| ER-0,39 | AITC-0,39 | 7,004 | 0,002557 |
| ER-0,19 | PEITC-0,19 | 12,95 | 4,52E-10 |
| PEITC-3,125 | BITC-3,125 | 33,93 | 4,35E-10 |
| PEITC-3,125 | AITC-3,125 | 33,93 | 4,35E-10 |
| PEITC-1,56 | BITC-1,56 | 45,9 | 4,35E-10 |
| PEITC-1,56 | AITC-1,56 | 13,82 | 4,37E-10 |
| PEITC-0,78 | BITC-0,78 | 13,93 | 4,36E-10 |
| PEITC-0,78 | AITC-0,78 | 14,76 | 4,36E-10 |
| PEITC-0,39 | BITC-0,39 | 10,81 | 2,19E-08 |
| PEITC-0,39 | AITC-0,39 | 17,92 | 4,35E-10 |
| PEITC-0,19 | BITC-0,19 | 11,56 | 2,17E-09 |
| PEITC-0,19 | AITC-0,19 | 13,41 | 4,39E-10 |
| BITC-1,56 | AITC-1,56 | 32,08 | 4,35E-10 |
| BITC-0,39 | AITC-0,39 | 7,117 | 0,001887 |
| ***S. aureus* ATCC 6538** |  | **Q** | **p** |
| CH-3,125 | ER-3,125 | 14.81 | 4,36E-07 |
| CH-3,125 | PEITC-3,125 | 11.87 | 1,05E-06 |
| CH-1,56 | ER-1,56 | 29.45 | 4,35E-07 |
| CH-1,56 | PEITC-1,56 | 29.67 | 4,35E-07 |
| CH-1,56 | BITC-1,56 | 10.63 | 3,85E-05 |
| CH-1,56 | AITC-1,56 | 34.42 | 4,35E-07 |
| CH-0,78 | ER-0,78 | 12.25 | 6,05E-07 |
| CH-0,78 | PEITC-0,78 | 11.06 | 9,67E-06 |
| CH-0,78 | BITC-0,78 | 6.766 | 0.004771 |
| CH-0,78 | AITC-0,78 | 11.91 | 9,63E-07 |
| CH-0,39 | ER-0,39 | 13.22 | 4,42E-07 |
| CH-0,39 | PEITC-0,39 | 12.79 | 4.63E-10 |
| CH-0,39 | BITC-0,39 | 15.76 | 4,36E-07 |
| CH-0,39 | AITC-0,39 | 12.97 | 4,51E-07 |
| CH-0,19 | ER-0,19 | 12.25 | 6,05E-07 |
| CH-0,19 | PEITC-0,19 | 16.16 | 4,36E-07 |
| CH-0,19 | BITC-0,19 | 11.69 | 1,56E-06 |
| CH-0,19 | AITC-0,19 | 9.397 | 2,12E-03 |
| ER-3,125 | BITC-3,125 | 14.81 | 4,36E-07 |
| ER-3,125 | AITC-3,125 | 14.81 | 4,36E-07 |
| ER-1,56 | BITC-1,56 | 18.82 | 4,35E-07 |
| PEITC-3,125 | BITC-3,125 | 11.87 | 1,05E-06 |
| PEITC-3,125 | AITC-3,125 | 11.87 | 1,05E-06 |
| PEITC-1,56 | BITC-1,56 | 19.04 | 4,35E-07 |
| PEITC-0,19 | AITC-0,19 | 6.766 | 0.004771 |
| BITC-1,56 | AITC-1,56 | 23.78 | 4,35E-07 |
| ***E. faecalis* ATCC 8043** |  | **Q** | **p** |
| CH-3,125 | ER-3,125 | 22.6 | 4,35E-07 |
| CH-3,125 | PEITC-3,125 | 30.73 | 4,35E-07 |
| CH-1,56 | ER-1,56 | 44.54 | 4,35E-07 |
| CH-1,56 | PEITC-1,56 | 56.39 | 4,35E-07 |
| CH-1,56 | BITC-1,56 | 33.82 | 4,35E-07 |
| CH-1,56 | AITC-1,56 | 60.15 | 4,35E-07 |
| CH-0,78 | ER-0,78 | 40.73 | 4,35E-07 |
| CH-0,78 | PEITC-0,78 | 53.79 | 4,35E-07 |
| CH-0,78 | BITC-0,78 | 38.47 | 4,35E-07 |
| CH-0,78 | AITC-0,78 | 46.17 | 4,35E-07 |
| CH-0,39 | ER-0,39 | 46.88 | 4,35E-07 |
| CH-0,39 | PEITC-0,39 | 58.56 | 4,35E-07 |
| CH-0,39 | BITC-0,39 | 50.27 | 4,35E-07 |
| CH-0,39 | AITC-0,39 | 42.57 | 4,35E-07 |
| CH-0,19 | ER-0,19 | 42.49 | 4,35E-07 |
| CH-0,19 | PEITC-0,19 | 53.79 | 4,35E-07 |
| CH-0,19 | BITC-0,19 | 42.82 | 4,35E-07 |
| CH-0,19 | AITC-0,19 | 41.06 | 4,35E-07 |
| ER-3,125 | PEITC-3,125 | 8.121 | 0.0001083 |
| ER-3,125 | BITC-3,125 | 22.6 | 4,35E-07 |
| ER-3,125 | AITC-3,125 | 22.6 | 4,35E-07 |
| ER-1,56 | PEITC-1,56 | 11.85 | 1,10E-06 |
| ER-1,56 | BITC-1,56 | 10.72 | 2,94E-05 |
| ER-1,56 | AITC-1,56 | 15.61 | 4,36E-07 |
| ER-0,78 | PEITC-0,78 | 13.06 | 4,47E-07 |
| ER-0,39 | PEITC-0,39 | 11.68 | 1.6E-09 |
| ER-0,19 | PEITC-0,19 | 11.3 | 4,55E-06 |
| PEITC-3,125 | BITC-3,125 | 30.73 | 4,35E-07 |
| PEITC-3,125 | AITC-3,125 | 30.73 | 4,35E-07 |
| PEITC-1,56 | BITC-1,56 | 22.56 | 4,35E-07 |
| PEITC-0,78 | BITC-0,78 | 15.32 | 4,36E-07 |
| PEITC-0,78 | AITC-0,78 | 7.619 | 0.0004677 |
| PEITC-0,39 | BITC-0,39 | 8.288 | 6,57E-02 |
| PEITC-0,39 | AITC-0,39 | 15.99 | 4,36E-07 |
| PEITC-0,19 | BITC-0,19 | 10.97 | 1,30E-05 |
| PEITC-0,19 | AITC-0,19 | 12.73 | 4,70E-07 |
| BITC-1,56 | AITC-1,56 | 26.33 | 4,35E-07 |
| BITC-0,78 | AITC-0,78 | 7.702 | 0.0003681 |
| BITC-0,39 | AITC-0,39 | 7.702 | 0.0003681 |

**Table S2.** Differences evidenced among every concentration of ITC solutions and the respective controls. P (p < 0.005) and Q values were evaluated with Tuckey post-hoc and two-way ANOVA. Gray rows evidenced the no statistically significant results of interactions. *Q=adj p value*

| ***E. coli* ATCC 8739** |  | **Q** | **p** |
| --- | --- | --- | --- |
| CH-25 | CH-0 | 67,21 | 4,35E-10 |
| CH-12,5 | CH-0 | 67,21 | 4,35E-10 |
| CH-6,25 | CH-0 | 67,21 | 4,35E-10 |
| CH-3,125 | CH-0 | 67,21 | 4,35E-10 |
| CH-1,56 | CH-0 | 67,21 | 4,35E-10 |
| CH-0,78 | CH-0 | 38,6 | 4,35E-10 |
| CH-0,39 | CH-0 | 27,71 | 4,35E-10 |
| CH-0,19 | CH-0 | 11,3 | 4,63E-09 |
| ER-25 | ER-0 | 74,78 | 4,35E-10 |
| ER-12,5 | ER-0 | 74,78 | 4,35E-10 |
| ER-6,25 | ER-0 | 74,78 | 4,35E-10 |
| ER-1,56 | ER-0 | 17,1 | 4,35E-10 |
| ER-0,78 | ER-0 | 5,912 | 0,03738 |
| ER-0,39 | ER-0 | 4,33 | 0,533 |
| ER-0,19 | ER-0 | 0,8284 | 1 |
| PEITC-25 | PEITC-0 | 89,02 | 4,35E-10 |
| PEITC-12,5 | PEITC-0 | 89,02 | 4,35E-10 |
| PEITC-6,25 | PEITC-0 | 89,02 | 4,35E-10 |
| PEITC-3,125 | PEITC-0 | 55,09 | 4,35E-10 |
| PEITC-1,56 | PEITC-0 | 18,15 | 4,35E-10 |
| PEITC-0,78 | PEITC-0 | 15,29 | 4,36E-10 |
| PEITC-0,39 | PEITC-0 | 7,644 | 0,0004349 |
| PEITC-0,19 | PEITC-0 | 2,109 | 1 |
| BITC-25 | BITC-0 | 80,13 | 4,35E-10 |
| BITC-12,5 | BITC-0 | 80,13 | 4,35E-10 |
| BITC-6,25 | BITC-0 | 80,13 | 4,35E-10 |
| BITC-3,125 | BITC-0 | 80,13 | 4,35E-10 |
| BITC-1,56 | BITC-0 | 55,16 | 4,35E-10 |
| BITC-0,78 | BITC-0 | 20,33 | 4,35E-10 |
| BITC-0,19 | BITC-0 | 4,782 | 0,3041 |
| AITC-25 | AITC-0 | 75,08 | 4,35E-10 |
| AITC-12,5 | AITC-0 | 75,08 | 4,35E-10 |
| AITC-6,25 | AITC-0 | 75,08 | 4,35E-10 |
| AITC-3,125 | AITC-0 | 75,08 | 4,35E-10 |
| AITC-1,56 | AITC-0 | 18,04 | 4,35E-10 |
| AITC-0,78 | AITC-0 | 16,12 | 4,36E-10 |
| AITC-0,19 | AITC-0 | 1,582 | 1 |
| ***S. aureus* ATCC 6538** |  | **Q** | **p** |
| CH-25 | CH-0 | 37.41 | 4,35E-07 |
| CH-12,5 | CH-0 | 37.41 | 4,35E-07 |
| CH-6,25 | CH-0 | 37.41 | 4,35E-07 |
| CH-3,125 | CH-0 | 37.41 | 4,35E-07 |
| CH-1,56 | CH-0 | 37.41 | 4,35E-07 |
| CH-0,78 | CH-0 | 14.95 | 4,36E-07 |
| CH-0,39 | CH-0 | 12.52 | 5,04E-07 |
| CH-0,19 | CH-0 | 7.149 | 0.001731 |
| ER-25 | ER-0 | 43.32 | 4,35E-07 |
| ER-12,5 | ER-0 | 43.32 | 4,35E-07 |
| ER-6,25 | ER-0 | 43.32 | 4,35E-07 |
| ER-3,125 | ER-0 | 28.5 | 4,35E-07 |
| ER-1,56 | ER-0 | 13.87 | 4,36E-07 |
| ER-0,78 | ER-0 | 8.61 | 2,48E-02 |
| ER-0,39 | PEITC-0,39 | 0.4271 | 1 |
| ER-0,19 | ER-0 | 0.8093 | 1 |
| PEITC-25 | PEITC-0 | 48.24 | 4,35E-07 |
| PEITC-12,5 | PEITC-0 | 48.24 | 4,35E-07 |
| PEITC-6,25 | PEITC-0 | 48.24 | 4,35E-07 |
| PEITC-3,125 | PEITC-0 | 36.37 | 4,35E-07 |
| PEITC-1,56 | PEITC-0 | 18.57 | 4,35E-07 |
| PEITC-0,78 | PEITC-0 | 14.72 | 4,36E-07 |
| PEITC-0,39 | PEITC-0 | 10.57 | 4,81E-05 |
| PEITC-0,19 | PEITC-0 | 1.821 | 1 |
| BITC-25 | BITC-0 | 42.85 | 4,35E-07 |
| BITC-12,5 | BITC-0 | 42.85 | 4,35E-07 |
| BITC-6,25 | BITC-0 | 42.85 | 4,35E-07 |
| BITC-3,125 | BITC-0 | 42.85 | 4,35E-07 |
| BITC-1,56 | BITC-0 | 32.21 | 4,35E-07 |
| BITC-0,78 | BITC-0 | 13.62 | 4,37E-07 |
| BITC-0,39 | BITC-0 | 2.203 | 1 |
| BITC-0,19 | BITC-0 | 0.8992 | 1 |
| AITC-25 | AITC-0 | 41 | 4,35E-07 |
| AITC-12,5 | AITC-0 | 41 | 4,35E-07 |
| AITC-6,25 | AITC-0 | 41 | 4,35E-07 |
| AITC-3,125 | AITC-0 | 41 | 4,35E-07 |
| AITC-1,56 | AITC-0 | 6.587 | 0.00755 |
| AITC-0,78 | AITC-0 | 6.632 | 0.00674 |
| AITC-0,19 | AITC-0 | 1.349 | 1 |
| ***E. faecalis* ATCC 8043** |  | **Q** | **p** |
| CH-25 | CH-0 | 47.09 | 4,35E-07 |
| CH-12,5 | CH-0 | 47.09 | 4,35E-07 |
| CH-6,25 | CH-0 | 47.09 | 4,35E-07 |
| CH-3,125 | CH-0 | 47.09 | 4,35E-07 |
| CH-1,56 | CH-0 | 47.09 | 4,35E-07 |
| CH-0,78 | CH-0 | 27.84 | 4,35E-07 |
| CH-0,39 | CH-0 | 15.28 | 4,36E-07 |
| CH-0,19 | CH-0 | 4.981 | 0.2245 |
| ER-25 | ER-0 | 85.6 | 4,35E-07 |
| ER-12,5 | ER-0 | 85.6 | 4,35E-07 |
| ER-6,25 | ER-0 | 85.6 | 4,35E-07 |
| ER-3,125 | ER-0 | 63 | 4,35E-07 |
| ER-1,56 | ER-0 | 41.06 | 4,35E-07 |
| ER-0,78 | ER-0 | 25.62 | 4,35E-07 |
| ER-0,39 | ER-0 | 6.907 | 0.003306 |
| ER-0,19 | ER-0 | 1.005 | 1 |
| PEITC-25 | PEITC-0 | 97.37 | 4,35E-07 |
| PEITC-12,5 | PEITC-0 | 97.37 | 4,35E-07 |
| PEITC-6,25 | PEITC-0 | 97.37 | 4,35E-07 |
| PEITC-3,125 | PEITC-0 | 66.64 | 4,35E-07 |
| PEITC-1,56 | PEITC-0 | 40.98 | 4,35E-07 |
| PEITC-0,78 | PEITC-0 | 24.32 | 4,35E-07 |
| PEITC-0,39 | PEITC-0 | 6.991 | 0.002648 |
| PEITC-0,19 | PEITC-0 | 1.465 | 1 |
| BITC-25 | BITC-0 | 85.9 | 4,35E-07 |
| BITC-12,5 | BITC-0 | 85.9 | 4,35E-07 |
| BITC-6,25 | BITC-0 | 85.9 | 4,35E-07 |
| BITC-3,125 | BITC-0 | 85.9 | 4,35E-07 |
| BITC-1,56 | BITC-0 | 52.07 | 4,35E-07 |
| BITC-0,78 | BITC-0 | 28.17 | 4,35E-07 |
| BITC-0,39 | BITC-0 | 3.809 | 0.8034 |
| BITC-0,19 | BITC-0 | 0.9628 | 1 |
| AITC-25 | AITC-0 | 84.77 | 4,35E-07 |
| AITC-12,5 | AITC-0 | 84.77 | 4,35E-07 |
| AITC-6,25 | AITC-0 | 84.77 | 4,35E-07 |
| AITC-3,125 | AITC-0 | 84.77 | 4,35E-07 |
| AITC-1,56 | AITC-0 | 24.61 | 4,35E-07 |
| AITC-0,78 | AITC-0 | 19.34 | 4,35E-07 |
| AITC-0,39 | AITC-0 | 10.38 | 8,78E-05 |
| AITC-0,19 | AITC-0 | 1.591 | 1 |
